# Supplementary figures and images for: Importance of experimental information (metadata) for archived sequence data: case of specific gene bias due to lag time between sample harvest and RNA protection in RNA sequencing
Source: PeerJ. 2021 Aug 25;9:e11875. doi: 10.7717/peerj.11875 (PMC8401820; doi:10.7717/peerj.11875)

**Atf4**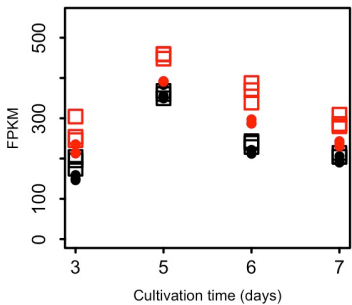**Btg2**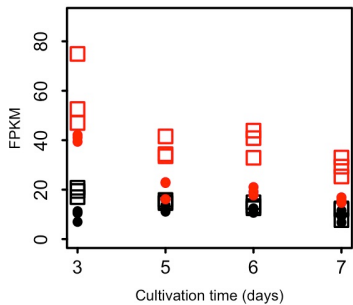**Chub2**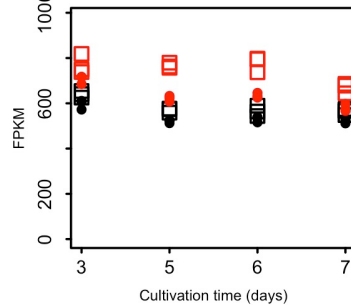**Cyr61**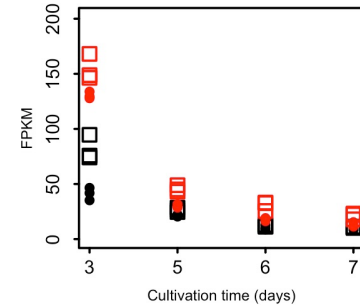**Ddx5**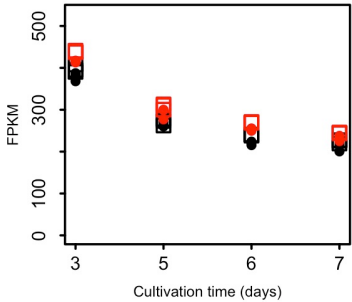**Dusp5**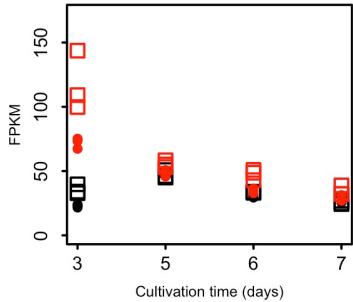**Egr1**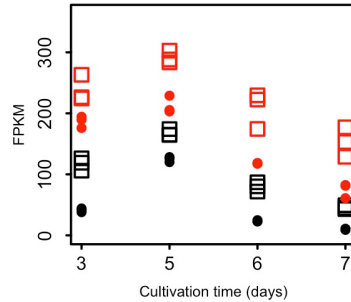**Egr2**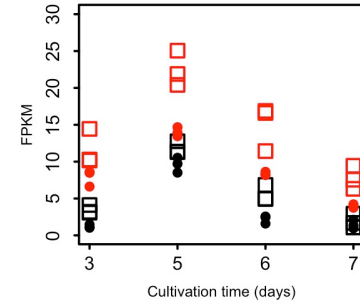**Fos**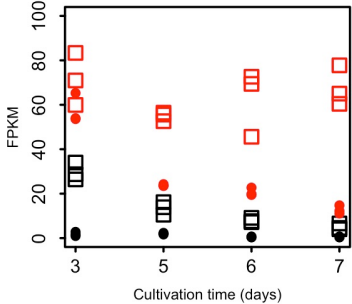**Fosb**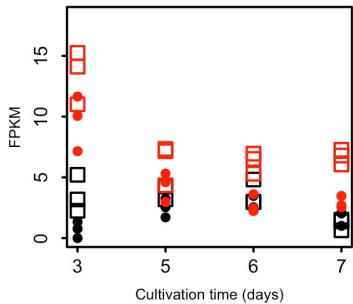**H3f3b**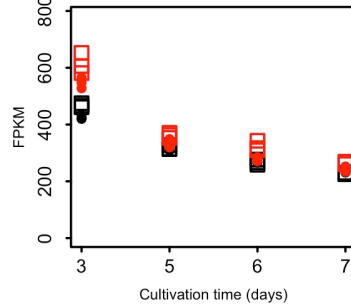**Ier3**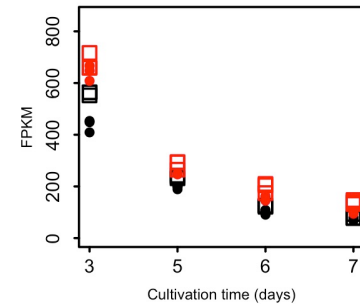**Ier5**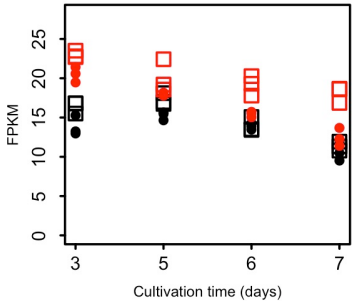**Jun**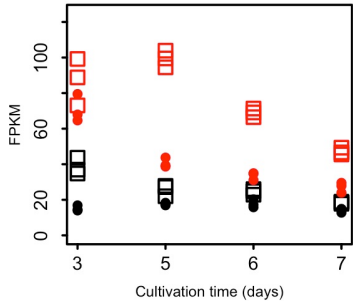**Junb**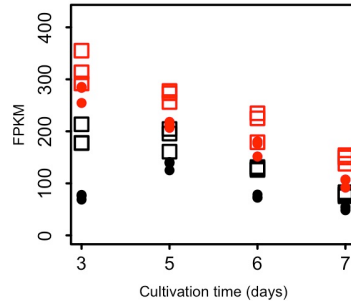**Klf6**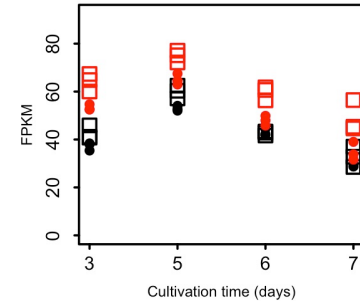**LOC103159497**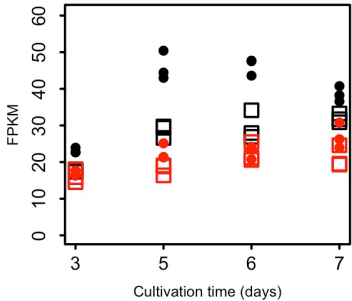**Plk2**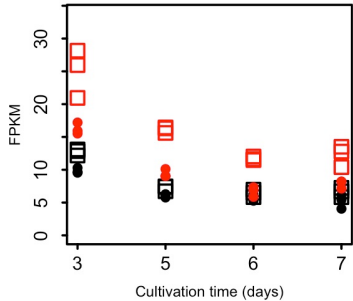**Ppp1r15a**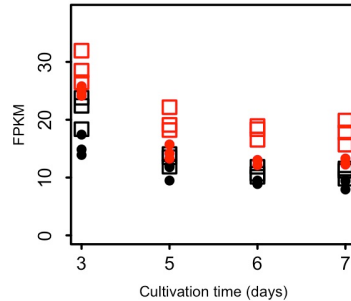**Sgk1**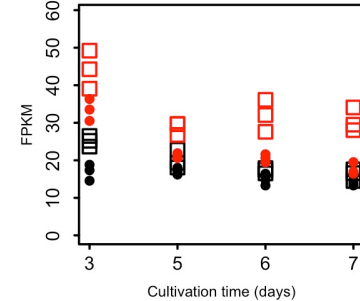**Srsf5**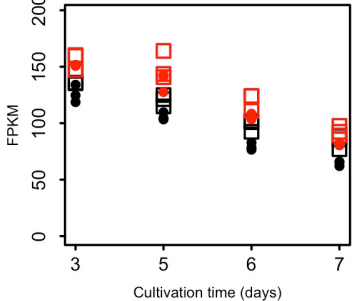**Vegfa**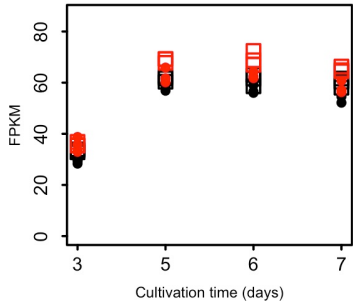**Zfp36**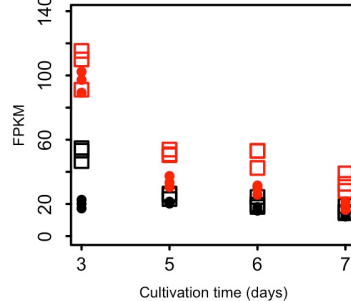

Supplement: Supplemental Information 1 — Black filled circles, black unfilled squares, red filled circles, and red unfilled squares indicate the processing lag times of 15, 30, 45, and 60 min, respectively. [file peerj-09-11875-s001.pdf]
